# Relationship Between Personality Types in MBTI and Dream Structure Variables

**DOI:** 10.3389/fpsyg.2020.01589

**Published:** 2020-08-12

**Authors:** Chuanwen Zhao, Jiaxi Wang, Xiaoling Feng, Heyong Shen

**Affiliations:** School of Psychology, South China Normal University, Guangzhou, China

**Keywords:** dream content, dream incorporation, dream metaphor, intuition, MBTI, MADRE

## Abstract

This study aimed to explore relationships between personality type variables and dream structure variables. In the questionnaire experiment (*N* = 410), we investigated associations between different personality variables in the Myers-Briggs Type Indicator questionnaire (MBTI) and various aspects of dreams in the Mannheim Dream questionnaire (MADRE). The MBTI has four dimensions. In the Extroversion/Introversion (E/I) dimension, I types dreamt more of emotional intensity and passive emotions than E types. In addition, I types may become more distressed in nightmares than E types. E types more frequently shared their dreams with others. In the Sensation/Intuition (S/N) dimension, N types had a more positive attitude toward dreams and can get more novel ideas and help from their dreams than S types. In the dream diary experiment (*N* = 47), we investigated whether the S/N dimension may influence waking events’ incorporation into dreams. External judges decoded paired waking events and dream reports. N types had more metaphorical incorporation than S types. More specifically, N types had more metaphorical expressions in their dreams than S types. This result may be due to the different characteristics between S types and N types. It may provide support for the dream continuity hypothesis.

## Introduction

Autobiographical memory (AM) is a “memory for the events of one’s life,” including personal semantic information and personal episodic information (Baddeley, 1992). Horton and her colleague use the AM experiences to explain the construction of dreams (Horton and Malinowski, 2015). According to their proposal, AM experiences are broken down into constituent fragments, reactivated “offline” during sleep, and re-combined into a novel experience. Dreams may reflect this process.

Empirical evidence has indicated that some factors can influence the incorporation of a waking life element into dreams (for a review see Horton and Malinowski, 2015), e.g., the dream’s emotionality (e.g., Malinowski and Horton, 2014) and when the dream happened (Blagrove et al., 2011a, b; Van et al., 2015). However, only a few studies explored the correlation between personality and the incorporation of waking events into dreams (e.g., Aumann et al., 2012). More empirical studies should therefore be designed to investigate this.

Jung classified personality types by three dimensions (Jung, 1921/1971, p. 540): Introversion (I)/Extroversion (E), Sensation (S)/Intuition (N) and Thinking (T)/Feeling (F).

“Extroversion means directing interest and attention toward people and things in the external world, whereas Introversion means directing interest and attention toward internal experiences and processes. Sensation means perceiving the presence and qualities of facts directly through the senses, whereas Intuition means perceiving the relations and possibilities in events and situations unconsciously or subliminally. Thinking means organizing and judging experience on the basis of analysis and logical meaning, whereas Feeling means organizing and judging experience on the basis of importance, values, likes and dislikes” (Jung, 1921/1971, p. 540; as cited in Cann and Donderi, 1986).

The Myers-Briggs Type Indicator (MBTI) has been developed to enable researchers to measure Jung’s psychological types (e.g., Myers, 1962; Carlson, 1985; Myers et al., 1998). It can help to measure Jung’s three personality dimensions (E/I, S/N, and T/F), and also a dimension proposed by Myers [judging (J)/perceiving (P)]. Judging and Perceiving are two processes by which we perceive and then act upon information; Perceiving is concerned with directly receiving information without evaluation and Judging is concerned with organizing and processing information.

Cann and Donderi (1986) studied the relationship between Jung’s personality types and Jung’s dream types (e.g., Jung, 1921/1971, 1948a, b). When defining the archetypal dreams as those filled with irrational, heightened effect, and mythological parallels, they found that N types had more archetypal dreams than S types. Some researchers noted that dreams could be metaphors for waking life, picturing waking-life experiences and emotions in non-literal, figurative ways (e.g., Lakoff, 1993; Hartmann, 1996; Domhoff, 2003). So archetypal dreams defined in Cann and Donderi (1986) may relate to dreams filled with metaphors of waking experiences. Wang and Shen (2018) established operational definitions for two kinds of waking events’ incorporation into dreams: the descriptive incorporation and the metaphorical incorporation. The former means a direct continuity between waking events and dreams, while the latter means an indirect continuity between waking events and dreams. Therefore, dreams filled with metaphors of waking experiences may be more likely to relate to the metaphorical incorporation, rather than the descriptive incorporation. So archetypal dreams may correlate to the metaphorical incorporation, rather than the descriptive incorporation. As Cann and Donderi (1986) found that N types had more archetypal dreams than S types, N types may have more metaphorical incorporation in their dreams than S types. In this study, one of our aims were to test this hypothesis.

In addition, a previous study investigated the relationship between the big five personality dimensions and dream variables (Aumann et al., 2012). The results showed that there were some correlations between personality traits of the big five and dream structure variables (e.g., the incorporation of waking events into dreams). However, Malinowski (2015) did not show any significant correlation between personality traits of the big five and participants reported of dreaming of overlaps with waking life experiences. So, which dream variables may correlate with personality traits of the big five deserved further study. There is evidence suggesting that the four MBTI indices measure aspects of four of the big five dimensions of personality (e.g., Furnham, 1996; Furnham et al., 2003). So, any potential significant correlations between personality dimensions of the MBTI and dream variables may imply significant correlations between personality traits of the big five and dream variables. To our knowledge, so far, no study has explored the correlation between personality dimensions of the MBTI and dream variables. We, therefore, here, made a survey to investigate this. The Mannheim Dream questionnaire (MADRE) can help to measure different aspects of dreams (Schredl et al., 2014), including dream recall frequency, dream emotion aspects (intensity/tone), different dream types, attitude toward dreams, what dreamers do with their dream (telling/recording), effects of dreams on waking life (creative dreams, problem solving dreams, déjà vu experiences based on dreams). In the present study, we used this questionnaire to measure dream structure variables.

Overall, the research had two purposes: one was to explore whether the S/N dimension may influence the incorporation of waking events into dreams. The other was to explore the relationship between personality dimensions of the MBTI and dream structure variables. We used a dream diary method to test our hypothesis and used a dream questionnaire method to explore the relationship.

## Materials and Methods

### Participants

410 people took part in the dream questionnaire experiment. They were either undergraduates or postgraduates at South China Normal University. 105 males and 305 females, with an average age 22.00, SD = 3.75, from 18 to 40, took part in the experiment. They finished the online dream questionnaire. In addition, 50 (25 N types\25 S types) of them also took part in the dream diary experiment. They were self-reported frequent dream recallers (recalling dreams several times a week or almost every day, measured by MADRE) and did not take recreational drugs, did not consume alcohol (measured by an online questionnaire), and had no sleep disorders or neurological/psychiatric history (measured by an online questionnaire). Of the 50 participants, three participants were removed from the analysis because they did not record their waking events and dreams on time every day. Results are thus from the remaining 47 participants (22 N types\25 S types; Male 5, Female 42; average age 21.18, SD = 1.83, from 18 to 27). Additionally, 3 months later, 30 of them volunteered to re-answer the MBTI questionnaire. Participants gave written informed consent before their experiments. This study was approved by the Research Ethics Committee of South China Normal University.

### Materials

#### The Dream Questionnaire Experiment

Both the MBTI-M questionnaire (93 items) and the MADRE questionnaire (28 items) composed the online questionnaire.

##### MBTI questionnaire

The Form M instrument is a reliable version of the MBTI assessment, with Cronbach alpha coefficients, from 0.91 to 0.92 (E/I, S/N, T/F, J/P; Myers et al., 1998). It also has good reliability across different samples (Schaubhut et al., 2009). The Chinese version of the MBTI-M has a high Cronbach alpha coefficient, from 0.70 to 0.87 (Cai et al., 2001). Here we used this Chinese version.

##### MADRE questionnaire

The MADRE questionnaire was devised to measure different aspects of dreams (Schredl et al., 2014), including dream recall frequency, dream emotion aspects (intensity/tone), different dream types, attitude toward dreams, what dreamers do with their dream (telling/recording), effects of dreams on waking life (creative dreams, problem-solving dreams, déjà vu experiences based on dreams). It consists of 28 items, eight items measure attitude toward dreams, and other items measure different aspects of dreams. Most of them showed high retest reliability (*r* = 0.70∼0.80), and a few items showed low reliability, with the lowest values being *r* = 0.585 (percentage of recurrent nightmares), and *r* = 0.617 (overall emotional tone of dreams); the retest coefficient of the total attitude toward dreams score was the highest (*r* = 0.842). Most of the items seem promising for psychometrics. Due to the language problem, the MADRE English version was translated into Chinese by two postgraduates and was directly used for psychological measurement purposes.

#### Dream Diary Experiment

##### Waking event collection

Waking events were divided into three categories, similar to Fosse et al. (2003) and Van et al. (2015): “Major daily activities (MDAs): Activities that took up most of the participants’ time during the day (e.g., going to work or university, meals, shopping). Personally significant events (PSEs): Important daily events that may or may not have taken up much time (e.g., emotional events). Major concerns (MCs): Concerns or thoughts that participants had on their mind during the day that may not have taken up much time, but were still considered important to them (e.g., money problems, exam stress).”

##### Dream collection

The requirements for recording a dream diary was same as that of a previous study (Selterman et al., 2012): “Describe everything in your dreams, with as much detail as possible: What happened, in what time frame, with whom, etc. Describe the cognitions, emotions, and behaviors you experienced in your dreams, as well as the cognitions, emotions and behaviors of all other parties included in your dreams (if evident to you). If it was a lucid dream, state so. Continue on the reverse side of this sheet if needed.”

##### Dream decoding

This coding system is taken from Wang and Shen (2018). Descriptive incorporation is the incorporation of conscious experiences into dreams in a direct way, while metaphorical incorporation is the incorporation of conscious experiences into dreams in an indirect way. Their operational definitions are outlined in Table 1.

**TABLE 1:** Operational definition for different kinds of incorporation.

Category	Operational definition
Descriptive incorporation	Dream subject element (e.g., character or object) is the same as the waking event’s description, and both the behavior^*b*^ and the behavioral outcome^*c*^ of the dream are in accord with the behavior^*b*^ and the behavior outcome^*c*^ of that event.
Metaphorical incorporation	(i) Dream subject element (e.g., character or object) is the same as the waking event’s description, and the dream’s behavior^*b*^ is not the same as the waking life behavior^*b*^ but their behavioral outcomes are the same as each other. (ii) Dream subject element (e.g., character or object) shares a similarity^*a*^ with the waking life event’s description, and the behavioral outcome^*c*^ of the dream is in accord with the behavior outcome^*c*^ of that event. If either i or ii can be found out, it would be viewed as metaphorical incorporation.

*^a^Similarity means that two elements can be categorized as of the same taxonomy (e.g., a character and an animal are viewed as the same taxonomy “creature,” while a character and a stone are not viewed as such). ^b^Behavior is the main action for a narrative event (e.g., for event “design for a home,” the behavior would be “design”). ^c^Behavioral outcome is the developmental consequence of a situation, usually producing either an advantage (e.g., to fulfill one’s desire, to solve a problem, etc.) or a disadvantage (e.g., cause a danger to, let someone down, etc.).*

### Procedure

In the dream questionnaire experiment, participants were asked to finish the online questionnaire. As a reward for this, they can get feedback on the MBTI test through E-mail. In the dream dairy experiment, participants recorded their dreams and waking experiences in a spreadsheet at home for 3 days. Specifically, participants recorded their waking events each evening and recorded their night dreams each morning (if they did not have dreams, they were required to record the words: no dreams), *via* a Chinese online questionnaire resource Wenjuanxing (similar to the online questionnaire resource Qualtrics). Dream dairies and waking experiences were paired by the same day (day events and that night’s dream). Finally, we obtained 121 paired events-dreams. Then, two blind external raters coded these events-dreams pairs (randomly arranged). The raters were the authors of this study. They scored each paired events-dreams by the operational definitions in Table 1. Considering MDAs seemed to have small incorporation in other studies (e.g., Malinowski and Horton, 2014; Van et al., 2015), external judges only scored PSEs and MCs.

### Data Analysis

#### Dream Questionnaire Experiment

In the MADRE questionnaire, the Cronbach alpha coefficients for the variable attitude toward dreams (8 items) was 0.911. We used the average score across the eight items to represent the variable attitude toward dreams. Other items were used, respectively. In the MBTI questionnaire, a forced-choice self-report measure of preferences on four bipolar dimensions was scored dichotomously. People with the higher score in one bipolar dimension would be dichotomized as one type in that dimension. People with the same score in one bipolar dimension would not be dichotomized as any type in that dimension.

#### Dream Diary Experiment

Two independent raters scored each type of incorporation for the total 121 paired events-dreams. Interrater reliability for judges’ initial rating scores was assessed. The Cronbach’s consistency coefficient was 0.73. All inconsistent ratings were later carefully discussed until an agreement was reached. The judges then scored each paired events-dreams having reached an agreement. A score of 0 for no presence or 1 for presence for each type of incorporation was used for the analysis. Scores of each type of incorporation of each participant were then averaged, and thus, each participant would provide one rating-score for each type of incorporation for further data analysis. All statistical analysis methods above were performed in IBM SPSS 18.0 software.

## Results

### Dream Questionnaire Experiment

#### Retest Reliability of MBTI

Pearson correlations showed that results of the MBTI retest reliability were high, from 0.837 to 0.908. The number of remaining participants and the retest reliability of in each categorical dimensions are provided in Table 2.

**TABLE 2:** The numbers of people and retest reliability in each categorical dimension.

Categorical dimension	Numbers of remained participants	Pearson correlation
Extroversion/Introversion	E 123/I 234	0.908***
Sensation/Intuition	S 187/N 183	0.837***
Thinking/Feeling	T 258/F 127	0.889***
Judging/Perception	J 275/P 116	0.845***

***p < 0.001.

#### Independent Sample *t*-Tests

E/I dimension: I types had more emotional intensity in their dreams than E types, *t* = 2.143, *p* = 0.035, Cohen’s *d* = 0.24; I types had a more passive emotional tone in their dreams than E types, *t* = −2.637, *p* = 0.09, Cohen’s *d* = 0.34; I types would become more distressed in nightmares than E types, *t* = 3.368, *p* = 0.001, Cohen’s *d* = 0.38; E types more often shared their dreams with others than I types, *t* = 3.011, *p* = 0.003, Cohen’s *d* = 0.34. The original data is provided in Table 3.

**TABLE 3:** The mean of different items in E/I variable.

Variable	Mean (SD)	
	Extroversion	Introversion
Nightmare distress^*a*^	2.51 (0.98)	2.88 (0.95)
Dream intensity^*b*^	2.46 (0.91)	2.68 (0.95)
Dream emotional tone^*c*^	3.02 (0.74)	2.81 (0.74)
Dream share frequency^*d*^	4.18 (1.78)	4.80 (1.90)

*^a^A five-point scale (1 = Not at all distressing, 2 = Not that distressing, 3 = Somewhat distressing, 4 = Quite distressing, and 5 = Very distressing). ^b^A five-point scale (1 = Not at all intense, 2 = Not that intense, 3 = Somewhat intense, 4 = Quite intense, 5 = Very intense). ^c^A five-point scale (1 = Very negative, 2 = Somewhat negative, 3 = Neutral, 4 = Somewhat positive, 5 = Very positive). ^d^An eight-point scale (1 = several times a week, 2 = about once a week, 3 = about 2 to 3 times a month, 4 = about once a month, 5 = about 2 to 4 times a year, 6 = about once a year, 7 = less than once a year, 8 = never).*

S/N dimension: N types had a more positive attitude toward dream than S types, *t* = 3.971, *p* < 0.001, Cohen’s *d* = 0.42; N types could more frequently get creative ideas from dreams than S types, *t* = −2.818, *p* = 0.005, Cohen’s *d* = 0.30; N types could more frequently get helps from dreams than S types, *t* = −2.1, *p* = 0.036, Cohen’s *d* = 0.22. The original data is provided in Table 4. T/F dimension and J/P dimension: Independent sample *t*-tests did not find any statistically significant difference in these dimensions.

**TABLE 4:** The mean of different items in S/N variable.

Variable	Mean (SD)	
	Sensation	Intuition
Attitude toward dream^*a*^	3.02 (0.79)	3.38 (0.93)
Creative ideas from dream^*b*^	6.23 (1.70)	5.70 (1.88)
Get helps from dream^*b*^	6.34 (1.67)	5.95 (1.92)

*^a^A five-point scale (1 = Not at all, 2 = Not that much, 3 = Partly, 4 = Somewhat, and 5 = Totally). an average number of 8 items. ^b^An eight-point scale (1 = several times a week, 2 = about once a week, 3 = about 2 to 3 times a month, 4 = about once a month, 5 = about 2 to 4 times a year, 6 = about once a year, 7 = less than once a year, 8 = never).*

### Dream Dairy Experiment

The analyzed data consists of 121 events-dreams pairs (58 from N types, and 63 from S types). For N types, the average length of a dream was 193.3 (SD = 155.2); the average number of PSEs was 1.52 (SD = 0.51), and the average number of MCs was 1.48 (0.65). For S types, the average length of a dream was 178.02 (SD = 136.37); the average number of PSEs was 1.53 (0.75); the average number of MCs was 1.53 (0.66). Independent sample *t*-tests showed that there was neither a Dream length difference nor an Events number difference between the two groups.

In addition, for N types, the frequency of non-incorporation was 43.1%, and the frequency of descriptive incorporation was 10.3%, and the frequency of metaphorical incorporation was 53.4%. By contrast, for S types, the frequency of non-incorporation was 55.6%, and the frequency of descriptive incorporation was 23.8%, and the frequency of metaphorical incorporation was 27%. Wilcoxon tests showed that N types had more metaphorical incorporation than S types (*Z* = 3.377, *p* = 0.001, effect size = 0.49, *n* = 47), and N types had less non-incorporation than S types (*Z* = 2.077, *p* = 0.038, effect size = 0.30, *n* = 47). There was no significant difference for descriptive incorporation between S types and N types (*Z* = 1.454, *p* = 0.146, *n* = 47).

## Discussion

In the dream questionnaire experiment, this study found some significant differences between personality type variables and dream structure variables, with small effect sizes (Cohen’s d from 0.22 to 0.42). These results are similar to Aumann et al. (2012) who also indicated a small correlation between personality variables and dream variables, e.g., except for the trait Neuroticism, other personality trait dimensions of the big five also obtained small correlations with dream variables.

In the E/I dimension: E types had a more positive emotional tone in their dreams than I types. This result was in accordance with previous work where people with a high extraversion score of NEO-FFI was found to have more positive emotions in their dreams (König et al., 2016), and this result was also partly in accordance with Aumann et al. (2012), who found that extraversion of NEO-FFI negatively correlated with aversive dream content (10 items, e.g., I dream of frightening events). Several studies have demonstrated the tendency for dreams to reflect emotional experiences from waking life (Schredl, 2006; Horton et al., 2011; Horton, 2012; Malinowski and Horton, 2014), which suggests that waking-life emotions tend to be incorporated into dreams. So, if people had more positive emotions in their waking life, then they would have more positive emotions in their dreams. Some studies found that extraversion was mainly linked with a positive mood (e.g., Meyer and Shack, 1989; Williams, 1990), and Verduyn and Brans (2012) indicated that the duration of positive emotions was the strongest predictor of extroversion, whereas the frequency of negative emotions was the strongest predictor of neuroticism. These implied that E types had more positive emotions in their waking life. Thus, E types could have a higher chance of dreaming about positive emotions. In assition, results showed that I types had a more emotional intensity of dreams than E types. A previous study showed that negative stimuli were experienced as more intense emotionally than positive stimuli on average (Ito et al., 1998). Since I types may have more negative emotions than E types, they could have more experiences with more emotional intensity in waking life. Experimental evidence showed that emotional experiences in waking-life were more preferential to be incorporated into dreams (e.g., Malinowski and Horton, 2014), thus I types could have a more emotional tone in their dream than E types. In addition, we found that I types might experience more nightmare distress than E types. This result was in line with Mcfatter (1998), who showed that extraversion was positively related to positive intensity, and also negatively related to negative intensity. Since the evidence showed that introverts exhibit higher reactivity to sensory stimulation than extraverts (for a short review see Stelmack, 1990), I types would be closer to passive emotions when they were in a bad situation. The nightmare was distressful, so in this situation, I types may become more distressed than E types. Finally, we found that that E types may more frequently share their dreams with others, which was in accordance with Schredl et al. (2016). This may be because extroverted people have more chances to talk with others, so they were more likely to tell their dreams to others.

In the S/N dimension: Results showed that N types could get more novel ideas from their dreams and get more help to recognize and solve problems from their dreams. Myers and Mccaulley (1985, p. 12) stated that MBTI intuition refers to “perception of possibilities, meaning and relationships by way of insight…[and] the unconscious. Intuitions may come to the surface of consciousness suddenly, as a “hunch,” the sudden perception of a pattern in seemingly unrelated events, or as creative discovery.” From this statement, we can see that N types may be more able to find correlations between the waking source domain and the dream domain. Previous evidence shows that consideration of the relationship between waking experiences and dreams can bring out insights and benefits (Hill et al., 1998; Edwards et al., 2013, 2015). So N types’ advantage to find correlations could give them more insights and benefits than S types. In addition, we also found that N types had a more positive attitude toward dreams than S types. This result may be because N types could get more benefits from their dreams than S types, as stated above, and thus the more benefits they got, the more positive attitudes they had toward dreams. These significant results of S/N dimension in the present study were in accordance with Aumann et al. (2012), who showed that the openness score positively correlated with the personal significance of dreams (13 items, e.g., my dreams give me advice).

In the dream diary experiment, we confirmed our hypothesis that N types had more metaphorical incorporation than S types. In the present study, the metaphorical incorporation was referred to as having more metaphorical expressions for waking events incorporated into dreams. The continuity hypothesis of dreaming proposes that the comparison of dream content with waking life suggests that dreams express one’s conceptions of the people and activities that concern them in waking life (e.g., Domhoff, 2003, 2011; for a critical review, see Domhoff, 2017). Pretz and Totz (2007) found that S/N dimension in MBTI uniquely measured the holistic nature of intuition as a preference for abstract and conceptual thought, in which holistic intuition meant a holistic judgment that integrated diverse sources of information. It was a Gestalt understanding of intuition, one that was qualitatively non-analytical. Thus, N types may have a greater tendency to use metaphorical expressions for the purpose to establish relationships between different domains in waking life. As a result, N types’ tendency to use more metaphorical expressions in waking life lead to them having more metaphorical incorporation than S types. This result gives support to the dream continuity hypothesis, and also implies that intuition is a personality factor that may influence the incorporation of waking events into dreams. In addition, we found that N types had less non-incorporation than S types. This result suggested that N types dreamed more of their waking events than S types. This result is partly in accordance with Aumann et al. (2012), who showed that openness was related to dream incorporation (13 items, e.g., I dream of people I met the preceding day).

### Method Consideration

The dream questionnaire experiment of the present study aimed to widely investigate personality traits and different aspects of dreams, which may provide more evidence for the correlation between other personality traits and various aspects of dreams. Though the MADRE questionnaire was translated into a Chinese version directly and without any revision, it may be enough to use, because all results were carried out by comparisons between different groups. This comparison may help to balance out potential inner errors of each group. In addition, all results were carefully compared with previous research, which may enhance their reliability.

The dream diary experiment of the present study aimed to make a comparison between personality types (N types and S types) and the incorporation of waking events into dreams. Previously, emotionality was found to affect the incorporation of waking events into dreams (e.g., Malinowski and Horton, 2014). As such, emotionality was important for the incorporation of waking events into dreams. However, in this study we did not ask participant to record the emotionality of their waking events. This may be a limitation of this study. Nevertheless, all participants were in the same college, and there was no evidence to suggest that intuition measured by MBTI was correlated with emotional trait variables such as the neuroticism; so, potential errors may be balanced out by the between-subject data analysis (N types versus S types). Future studies for potential repetitive purposes should control for the emotionality of waking events.

Furthermore, there is evidence suggesting that subsequent dreams reported after multiple awakenings of the same night, show both repeated incorporations of pre-sleep stimuli or suggestions and high frequencies of semantically equivalent or similar (so-called “interrelated”) contents (for a review, see Cipolli et al., 2016). In the present study, participants reported their dreams at home, so it was not clear whether the incorporation of waking events into dreams was caused by the number of awakenings of participants. Future studies should conduct a lab study to address this issue.

In addition, previously, Eichenlaub et al. (2014) found that differences in dream recall frequency were associated with differences in spontaneous brain activity in the temporoparietal and medial prefrontal cortex during both sleep and wakefulness. This result suggested that there was a neurophysiological basis for individual differences in dream variables. In the dream diary experiment, we only used the MBTI to measure a trait-like variable of participants, and then explored the correlation between the variable (intuition and sensation dimension) and the incorporation of waking events into dreams. This measure was a preliminary one. If possible, future studies should use neurophysiological methods (e.g., Polysomnography, PSG) to further investigate the potential individual differences of intuition (measured by MBTI) in the incorporation of waking events into dreams.

### Summary and Conclusion

This study explored relationships between personality type variables in MBTI and dream structure variables in MADRE, and also investigated relationships between S/N dimension and the incorporation of a waking life element into dreams. In the questionnaire experiment, results showed some significant differences with small effect-sizes (Cohen’s d from 0.22 to 0.42). Specifically, I types had a more emotional intensity and passive emotional tone in their dreams than E types. I types may become more distressed in nightmares than E types. E types more frequently shared their dreams with others. N types had a more positive attitude toward dreams and can get more novel ideas and help from their dreams than S types. Most of these results were in accordance with previous research and provided further evidence for them. In the dream diary experiment, we found that N types had more metaphorical incorporation than S types. More specifically, N types had more metaphorical expressions in their dreams than S types. This result can be explained by the individual difference of cognition tendency in waking life between N types and S types and may provide support to the dream continuity hypothesis.

## Data Availability Statement

All datasets generated for this study are included in the article/supplementary material.

## Ethics Statement

The studies involving human participants were reviewed and approved by the Ethics Committee in South China Normal University. The patients/participants provided their written informed consent to participate in this study.

## Author Contributions

CZ and JW: design. CZ: data collection. JW and XF: data analysis. CZ and JW: manuscript. HS: editor. All authors contributed to the article and approved the submitted version.

## Conflict of Interest

The authors declare that the research was conducted in the absence of any commercial or financial relationships that could be construed as a potential conflict of interest.
